# Comparative Evaluation of TRAIL, FGF-2 and VEGF-A-Induced Angiogenesis In Vitro and In Vivo

**DOI:** 10.3390/ijms17122025

**Published:** 2016-12-02

**Authors:** Siân P. Cartland, Scott W. Genner, Amna Zahoor, Mary M. Kavurma

**Affiliations:** 1 Heart Research Institute, Sydney 2042, Australia; sian.cartland@hri.org.au (S.P.C.); scott.genner@hri.org.au (S.W.G.); amna.zahoor@alumni.uts.edu.au (A.Z.); 2 Sydney Medical School, University of Sydney, Sydney 2006, Australia

**Keywords:** angiogenesis, proliferation, scratch assay, tubule formation, Matrigel plug, TRAIL, VEGF-A, FGF-2

## Abstract

Tumor necrosis-factor-related apoptosis-inducing ligand (TRAIL) has been implicated in angiogenesis; the growth of new blood vessels from an existing vessel bed. Our aim was to compare pro-angiogenic responses of TRAIL, vascular endothelial growth-factor-A (VEGF-A) and fibroblast growth-factor-2 (FGF-2) either separately (10 ng/mL) or in combination, followed by the assessment of proliferation, migration and tubule formation using human microvascular endothelial-1 (HMEC-1) cells in vitro. Angiogenesis was also measured in vivo using the Matrigel plug assay. TRAIL and FGF-2 significantly augmented HMEC-1 cell proliferation and migration, with combination treatment having an enhanced effect on cell migration only. In contrast, VEGF-A did not stimulate HMEC-1 migration at 10 ng/mL. Tubule formation was induced by all three factors, with TRAIL more effective compared to VEGF-A, but not FGF-2. TRAIL at 400 ng/mL, but not VEGF-A, promoted CD31-positive staining into the Matrigel plug. However, FGF-2 was superior, stimulating cell infiltration and angiogenesis better than TRAIL and VEGF-A in vivo. These findings demonstrate that each growth factor is more effective at different processes of angiogenesis in vitro and in vivo. Understanding how these molecules stimulate different processes relating to angiogenesis may help identify new strategies and treatments aimed at inhibiting or promoting dysregulated angiogenesis in people.

## 1. Introduction

Angiogenesis is the growth of new blood vessels from a pre-existing vessel bed, an essential process in the healthy body for development, wound healing and restoring blood flow and oxygen to tissues after injury. It is a tightly controlled process regulated by hypoxia and other mediators, including vascular endothelial growth factor (VEGF), fibroblast growth factor-2 (FGF-2), epidermal growth factor (EGF), platelet-derived growth factor (PDGF) and insulin-like growth factor 1 receptor (IGF1R) [1]. Angiogenesis generally involves activation, migration and proliferation of endothelial cells (EC) that ultimately assemble into solid cords or tubules, acquire a lumen and attach to existing vessels. In the maturation phase, vascular smooth muscle cells (VSMCs) and pericytes encapsulate and stabilize the vessel, affecting tone and blood flow in the newly-formed vessel. Uncontrolled angiogenesis, either excessive or insufficient, is increasingly associated with the pathogenesis of many chronic diseases, including tumor growth and metastasis, diabetes and cardiovascular diseases [1].

Tumor necrosis factor-related apoptosis-inducing ligand (TRAIL) is a molecule that regulates both cell survival and apoptosis; and because of this, the role of TRAIL in angiogenesis has been unclear. For example, TRAIL can inhibit VEGF-stimulated angiogenesis by inducing EC death [2]. TRAIL can also promote microvessel growth in vitro by increasing EC migration, invasion and proliferation and is pro-angiogenic, with activity comparable to VEGF in Matrigel assays for tubule formation [3]. We recently demonstrated that *Trail^−/−^* mice have reduced vascularity and angiogenesis in response to ischemic injury compared to the wildtype, and adenoviral delivery of TRAIL not only stimulated angiogenesis, but also improved blood flow to the lower limbs after hindlimb ischemia [4]. Interestingly, TRAIL is expressed in highly vascularized malignant mesenchymal tumors [3] suggesting that an association between TRAIL and vascularity in cancer exists; however, there is no direct evidence of how TRAIL may regulate angiogenesis in these processes.

In this report, our aim was to compare the angiogenic response of physiological concentrations of TRAIL, with the two most commonly-used angiogenic factors VEGF-A and FGF-2, at the same dose. Furthermore, we assessed the efficacy of the combination of all three factors in stimulating proliferation, migration and tubule formation in vitro and in vivo, using the Matrigel plug model. Here, we show that the pro-angiogenic responses by the three factors at 10 ng/mL is different; these findings may be useful in the design of new therapies aimed at inhibiting or activating angiogenesis.

## 2. Results

### 2.1. TRAIL Promotes HMEC-1 Proliferation to the Same Degree as FGF-2

**Figure 1:**
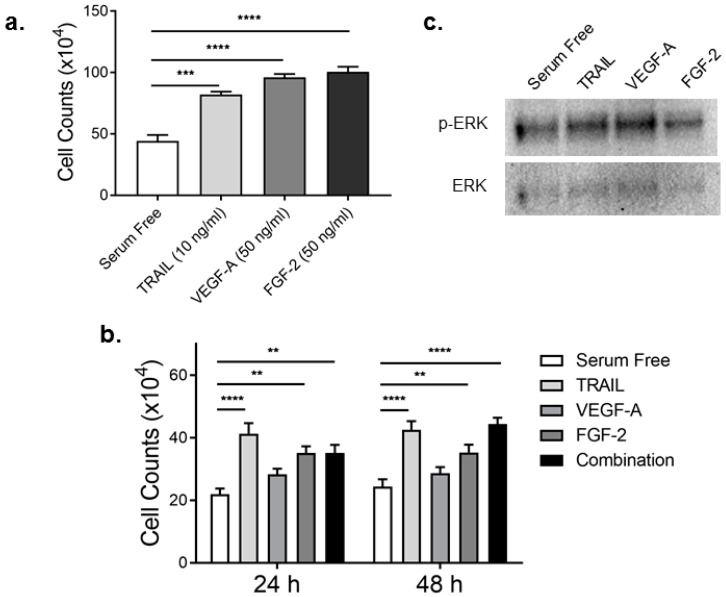
TRAIL and FGF-2 promote HMEC-1 cell proliferation. (**a**) Serum-starved HMEC-1 were exposed to TRAIL, VEGF-A and FGF-2 using indicated concentrations known to promote proliferation. Total cell counts were assessed 24 h later (*n* = 3/condition); (**b**) Serum-arrested HMEC-1 treated with TRAIL (10 ng/mL), VEGF-A (10 ng/mL), FGF-2 (10 ng/mL) or a combination of all three (each at 10 ng/mL). Cells were then assessed for total cell counts at 24 and 48 h (*n* = 3/condition); (**c**) ERK phosphorylation and expression. Serum-starved HMEC-1 were treated with each growth factor (10 ng/mL) for 15 min, followed by total protein extraction and Western blotting. Results are expressed as the mean ± SEM; ANOVA; ** *p* < 0.01, *** *p* < 0.001, **** *p* < 0.0001.

EC proliferation is a pivotal aspect of the tightly regulated process of angiogenesis [5]. VEGF-A and FGF-2 are established pro-angiogenic growth factors, known to promote EC proliferation [6]. TRAIL can also induce EC proliferation in vitro and in vivo [4]. To date, a comprehensive analysis of TRAIL, VEGF-A and FGF-2 or the effect of combination treatment on EC proliferation has not been examined. We treated HMEC-1 cells with 10 ng/mL TRAIL, 50 ng/mL VEGF-A and 50 ng/mL FGF-2, doses known to stimulate cell proliferation [4]. TRAIL at 10 ng/mL promoted HMEC-1 proliferation just as effectively as VEGF-A at 50 ng/mL (Figure 1a). In contrast, FGF-2’s ability to stimulate proliferation at 50 ng/mL was significantly increased compared to 10 ng/mL TRAIL (TRAIL vs. FGF-2: 82.17 ± 2.32 vs. 100.5 ± 4.16; *p* = 0.030; analysis of variance (ANOVA)). While this proved that all growth factors could promote HMEC-1 proliferation, whether a similar response was observed at the lower dose was examined. HMEC-1 proliferation in response to all growth factors at 10 ng/mL, as well as the combination of all three growth factors (10 ng/mL each) were assessed. Here, TRAIL significantly increased HMEC-1 proliferation at both 24 and 48 h ~1.9- and 1.8-fold, respectively (Figure 1b). Proliferation of HMEC-1 was also significantly elevated with 10 ng/mL FGF-2, ~1.6-fold at 24 h and 1.4-fold at 48 h (Figure 1b). VEGF-A at 10 ng/mL increased cell proliferation ~1.3-fold at 24 h; this was only significant when compared to serum free without multiple comparisons (serum free vs. VEGF-A: 22 ± 1.775 vs. 28.33 ± 1.789; *p* = 0.0198; *t-*test). Combination treatment did not have an additive or synergistic effect, but was comparable to TRAIL or FGF-2 alone (Figure 1b). Importantly, all three growth factors modestly stimulated ERK phosphorylation; the MAPK signaling pathway necessary for cell growth (Figure 1c). These findings suggest that 10 ng/mL TRAIL or FGF-2 promote HMEC-1 proliferation to the same degree.

### 2.2. TRAIL Promotes HMEC-1 Migration More Effectively than VEGF-A and FGF-2, with Combination Treatment Having Additive Effects

**Figure 2:**
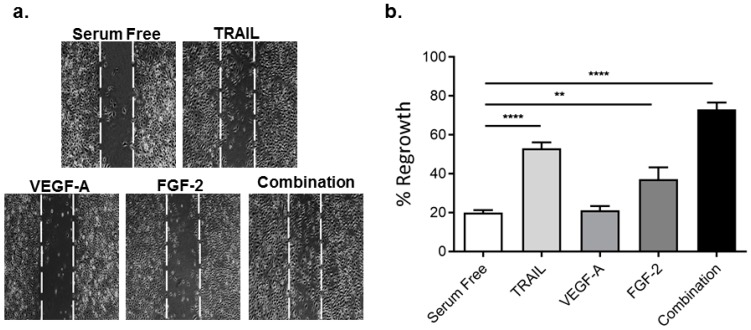
TRAIL and FGF-2 promote HMEC-1 migration. An in vitro scratch injury was performed in serum-arrested HMEC-1 cells followed by treatment with TRAIL (10 ng/mL), VEGF-A (10 ng/mL), FGF-2 (10 ng/mL) or the combination of all three (each at 10 ng/mL) as describe in the Methods. Images were taken 24 h later. (**a**) Representative images 10× magnification; the denuded zone is depicted by the space between the dashed lines; (**b**) quantification of cell migration (*n* = 3/condition) as described in the methods. Results are expressed as the mean ± SEM; ANOVA; ** *p* < 0.01, **** *p* < 0.0001.

Migration is an important step in the angiogenic response. Using an in vitro scratch assay, we assessed the effect of TRAIL, VEGF-A and FGF-2 on HMEC-1 migration into the wound area [4]. Exposure of HMEC-1 to TRAIL significantly augmented HMEC-1 migration into the denuded zone ~2.7-fold (Figure 2). FGF-2 also increased HMEC-1 migration ~1.9-fold, whereas VEGF-A had no effect at 10 ng/mL (Figure 2). Interestingly, combination treatment stimulated cell migration greater than single treatment alone (Figure 2). These findings suggest that TRAIL at 10 ng/mL stimulates HMEC-1 migration more effectively than VEGF-A and FGF-2, with combination therapy further augmenting migration.

### 2.3. TRAIL Promotes HMEC-1 Tubule Formation More Effectively than VEGF-A, but is Comparable to FGF-2 Treatment

**Figure 3:**
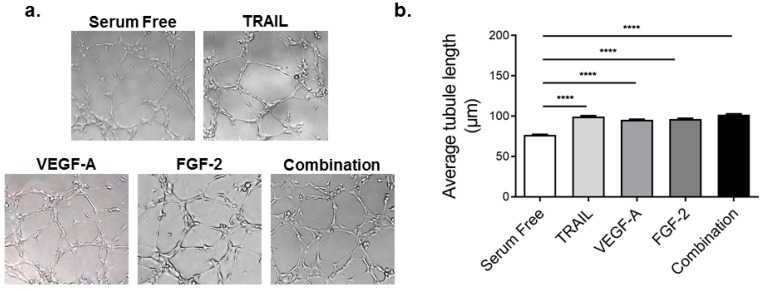
TRAIL promotes HMEC-1 tubule formation better than VEGF-A, yet comparable to FGF-2 and combination therapy. Serum-starved HMEC-1 cells were seeded into growth factor reduced Matrigel and treated with TRAIL (10 ng/mL), VEGF-A (10 ng/mL), FGF-2 (10 ng/mL) or the combination of all three (each at 10 ng/mL). Images were taken 24 h later. (**a**) Representative images 10× magnification; (**b**) tubule length was measured (*n* = 3/condition). Results are expressed as the mean ± SEM; ANOVA; **** *p* < 0.0001.

Tubule formation is a critical aspect of angiogenesis, representing one of the later stages [7]. Since EC differentiate and rapidly form tube-like structures when cultured on matrix basement membrane [8], tubule formation or tube-like structures can be measured in vitro [7]. Here, we measured tubule length as a marker of tubule formation in vitro. Compared to serum free, TRAIL, VEGF-A and FGF-2 (all at 10 ng/mL) significantly increased tubule length by 31%, 25% and 27%, respectively (Figure 3). Of note, TRAIL-stimulated HMEC-1 tubule formation was significantly greater than VEGF-A alone (99.51 ± 1.02 µm vs. 95.44 ± 0.90 µm; *p* < 0.05; *t*-test) (Figure 3). Combination treatment, however, did not further enhance tubule formation in HMEC-1 cells. Taken together, these findings demonstrate that tubule length is significantly augmented by all three growth factors independently, and combination treatment did not further enhance this response.

### 2.4. TRAIL Promotes the Infiltration of Cells and Increases EC Content in Matrigel Plugs

**Figure 4:**
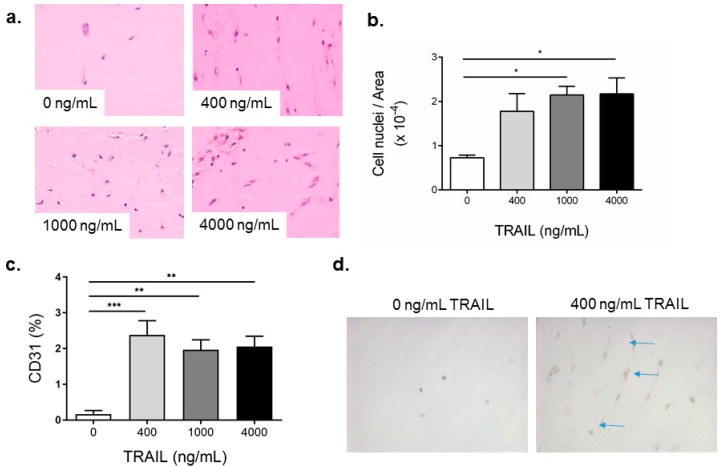
TRAIL promotes cell infiltration and increases EC content in Matrigel plugs. Liquid Matrigel containing increasing concentrations of TRAIL, as indicated, was injected subcutaneously into the flank of C57Bl/6J mice where it solidified and formed a plug. After 14 days, the plugs were excised, fixed, sectioned and assessed (**a**) by H&E for total cellular infiltration, with representative images and (**b**) quantification of total cell infiltration and (**c**) % CD31 staining in response to growth factors; (**d**) Representative images of CD31 staining (blue arrows) from 0 and 400 ng/mL TRAIL treated Matrigel plugs in mice. Images were taken at 40× magnification. Results are expressed as the mean ± SEM (*n* = 3 mice per condition); ANOVA; * *p* < 0.05, ** *p* < 0.01, *** *p* < 0.001.

We have previously shown TRAIL to promote angiogenesis in response to ischemia in the hind-limb of mice [4]. Both VEGF-A and FGF-2 are routinely used in Matrigel plug assays; however, whether TRAIL can promote angiogenesis into a plug in vivo is unknown. We first performed a dose response where Matrigel plugs containing 400, 1000 or 4000 ng/mL of TRAIL were injected into the flank of C57Bl/6 mice. Two weeks later, plugs were excised for histological analysis. C57Bl/6 mice administered vehicle only displayed minimal cellular infiltration (Figure 4a,b), with an increased number of cells in response to TRAIL at all concentrations (Figure 4a,b). CD31 was used as a marker to indicate vascularization or blood vessel formation within Matrigel plugs. As shown in Figure 4c,d, a significant increase in CD31-positive expression was observed in plugs at all concentrations of TRAIL. Importantly, the lowest dose of TRAIL was sufficient to promote CD31^+^ cell expression into the plug, which was comparable to 1000 and 4000 ng/mL TRAIL. These findings suggest that TRAIL significantly promotes blood vessel formation even at the lowest concentration, and the effect observed does not appear to be dose dependent.

### 2.5. FGF-2 Increases CD31-Positive Cells in the Matrigel Plug

**Figure 5:**
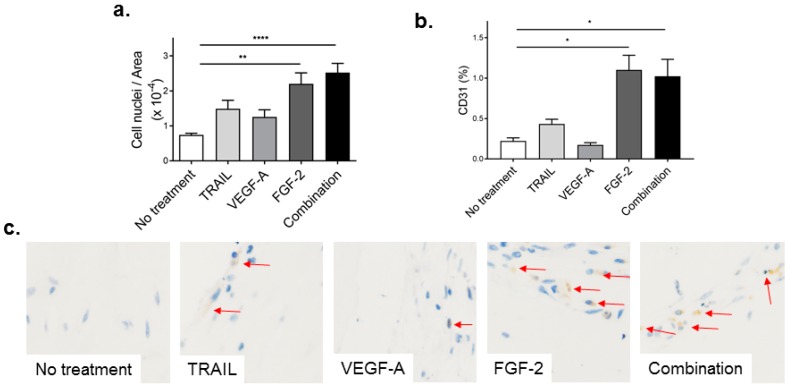
FGF-2 increases cell infiltration and CD31 capillaries in the Matrigel plug. Liquid Matrigel containing TRAIL, VEGF-A or FGF-2 at 400 ng/mL, or a combination of all three at 400 ng/mL each, was injected subcutaneously into the flank of C57Bl/6J mice where it solidified and formed a plug. After 14 days, plugs were excised, fixed, sectioned and assessed. (**a**) Quantification of cell infiltration into the Matrigel plug assessed from H&E sections; and (**b**) quantification of EC content by CD31 immunochemistry; (**c**) representative images of CD31 immunostaining (red arrows). All images were taken at 40× magnification. Results are expressed as the mean ± SEM (*n* = 6–7 mice per condition); ANOVA; * *p* < 0.05, ** *p* < 0.01, **** *p* < 0.0001.

Given that 400 ng/mL TRAIL were sufficient to promote cellular infiltration and CD31-positive staining, we compared the effect of 400 ng/mL TRAIL to VEGF-A, FGF-2 and combination treatment on angiogenesis using this concentration in vivo. Plugs containing single or a combination of growth factors (each at 400 ng/mL) had an increased number of cells compared to vehicle to variable degrees (Figure 5a; TRAIL, 102%; VEGF-A, 70%; FGF-2, 198%; combination, 241% compared to no treatment). Notably, a significant increase in cell infiltration was observed in mice administered Matrigel containing 400 ng/mL FGF-2 or a combination of all three factors (Figure 5a).

We next wanted to identify CD31-positive cells in the plug of all treatment groups. FGF-2 at 400 ng/mL significantly increased CD31-positive cell staining (Figure 5b). Importantly, TRAIL also augmented CD31-positive staining in the plug compared to no treatment (no treatment vs. TRAIL; 0.22 ± 0.04 vs. 0.43 ± 0.07, *p* = 0.03; *t*-test); however, when this was compared with all treatment groups, TRAIL had no effect (Figure 5b,c). In marked contrast, VEGF-A at 400 ng/mL did not alter CD31^+^ cell expression compared to the no treatment control at this dose (no treatment vs. VEGF-A; 0.22 ± 0.04 vs. 0.17 ± 0.03, *p* = 0.33; *t*-test). Taken together, these data suggest that FGF-2 at 400 ng/mL is superior at promoting blood vessel formation in the plug compared to TRAIL or VEGF-A.

## 3. Discussion

The angiogenic process involves multiple steps, including destabilization of existing blood vessels and degradation of the extracellular matrix; proliferation, sprouting and migration of EC towards an angiogenic stimulus; and the formation of tubular structures. In this report, we compared the angiogenic response of TRAIL, VEGF-A and FGF-2 in vitro and in vivo. First, in vitro, TRAIL promoted HMEC-1 proliferation and migration more than VEGF-A, but not FGF-2 at 10 ng/mL. Tubule length in response to TRAIL was also comparable to FGF-2, but increased compared to VEGF-A. In contrast, the combination of TRAIL, VEGF-A and FGF-2 treatment was more effective at stimulating the migration of HMEC-1. Second, in vivo, using the Matrigel model of angiogenesis, we identified that while 400 ng/mL TRAIL increased CD31^+^ cells into the plug, FGF-2 at the same concentration was superior. VEGF-A, at this dose had no effect. These findings are important since comparing the effect of different angiogenic factors to processes of angiogenesis both in vitro and in vivo is a crucial step in identifying new treatment options aimed at inhibiting or activating angiogenesis.

Angiogenesis is an important process in multiple clinical settings, including peripheral and coronary artery diseases, cancer, would healing, ocular diseases and many others. Therapeutics aimed at manipulating angiogenesis have great potential. The most commonly-described angiogenic growth factors are VEGF-A and FGF-2; both capable of stimulating EC proliferation, migration, sprouting and tubule formation [6,9,10]. Stimulation of angiogenesis, for example, could restore blood flow and oxygen to tissues after ischemic injury, particularly in the context of peripheral artery or coronary artery disease. VEGF, FGF-1 and -2 significantly improved the angiogenic response to ischemia by increasing the rate of perfusion recovery (reviewed in [1]). A cDNA encoding the VEGF-165 isoform administered to patients with severe ischemic heart disease improved exercise tolerance and reduced their symptoms [11]. A similar finding was observed for VEGF-2 [12,13] and FGF-2 [14] to a lesser extent. Bone marrow-derived mononuclear cells (MNCs) were shown to produce VEGF and FGF-2 [15], and MNC angiogenic therapy is now performed world-wide in patients with ischemic limbs [16] or injected into diseased myocardium [17]. TRAIL is also implicated in the process of angiogenesis [3,4]. More recently, we have shown that TRAIL adenovirus administration improved limb perfusion and stimulated capillary formation in ischemic muscle after hind-limb ischemic injury in mice [4]. Whether TRAIL could have potential therapeutic effects in ischemic disease in people is yet to be investigated. Nevertheless, attempting to rescue ischemic tissue by stimulation of new blood vessel growth is an important strategy to combat this problem.

On the contrary, inhibition of angiogenesis or anti-angiogenic therapy could be beneficial in oncology or in ocular diseases. Tumor angiogenesis is the generation of blood vessel networks within a cancerous growth, essential for supplying nutrients and critical for tumor growth and metastasis. Inhibiting angiogenesis would limit this supply to the growing tumor. In fact, anti-VEGF therapy has been FDA approved for metastatic colorectal cancer and non-small cell lung cancer [18]. Anti-VEGF treatment for ocular diseases, such as age-related macular degeneration, is also in use in the clinic today [19]. Whether TRAIL could also be a target for anti-angiogenic therapy is unknown.

Unlike VEGF-A and FGF-2, TRAIL’s effect on EC proliferation is conflicting given that it can stimulate the apoptosis or proliferation of EC. TRAIL at 100 ng/mL can induce apoptosis [2], while more physiological levels (~10 ng/mL) promote cell growth [3,4]. In this report, we show that 10 ng/mL TRAIL, FGF-2 and combination treatment significantly increased HMEC-1 proliferation compared to untreated cells. Interestingly, we found that TRAIL increased the proliferation of HMEC-1 more effectively than VEGF-A under the same conditions. Other studies using TRAIL and VEGF-A have seen similar effects. For example, 10 ng/mL TRAIL increased human umbilical cord EC (HUVEC) proliferation, with the combination of TRAIL and VEGF-A increasing cell proliferation in an additive manner [3]. We did not observe an additive response with HMEC-1. In fact, in this report, combination treatment displayed levels of proliferation that were comparable to TRAIL or FGF-2 alone.

Here, we also showed that TRAIL promoted EC migration to a greater degree than FGF-2 and VEGF-A. Remarkably, Cantarella et al [20] demonstrated TRAIL’s migratory effect on HUVEC at a concentration as low as 4 ng/mL, with VEGF-A having little effect on migration. In contrast to this study, we found that the combination of all three factors was even more effective at stimulating the migration of HMEC-1 into the denuded zone. However, the additive migratory response observed with combination treatment was probably not due to VEGF-A, but rather TRAIL and FGF-2. Collectively, differences in proliferation and migration between studies are likely due to the EC types used (HMEC-1 used in this study vs. HUVEC), given that EC from different vascular beds have different sensitivity to angiogenic growth factors [21].

Tubule formation is an in vitro model for assessing the differentiation of EC into tube-like structures, occurring subsequent to EC proliferation and migration. While VEGF-A and FGF-2 mediate EC tubule formation [6], these effects are seldom reported at low concentrations. TRAIL’s role in EC tubule formation is also conflicting. In this report, we showed TRAIL to stimulate HMEC-1 tubule formation more than VEGF-A alone. In direct contrast, EC tubule formation was inhibited by TRAIL, albeit TRAIL was used at a 20-fold higher concentration [2] compared to our study. The density and type of EC were also different, with Cantarella et al. [20] using 70,000 HUVEC/well. Variations in these aspects are known to influence tubule formation. For example, if the plating density is too high, EC can remain undifferentiated [8]. Moreover, we assessed tubule formation at 3 h after treatment rather than at 24 h [2]. It has been reported that tubules often start to deteriorate within 18 h of plating since EC undergo apoptosis [8]. By assessing tubule formation at 24 h, it may be probable that EC death may have occurred in this instance. Importantly, we showed all treatment groups to significantly augment tubule length. Of note, tubule length is only one of many measures used to assess tubule formation in vitro. Other common variables include the number of tubules, average tubule area, the number of closed networks and the number of branch points [8,22]. Different growth factors may also affect these variables differently and, as such, have significant implications for their use as pro-angiogenic agents [8].

Whether TRAIL treatment promoted angiogenesis better than or comparable to VEGF-A or FGF-2 treatment was assessed in vivo using a Matrigel plug assay. Importantly, we found that FGF-2 and combination treatment were more effective in increasing CD31-positive cells (and cellular infiltrate) into the plug. VEGF-A at 400 ng/mL may not have been the optimum dose to stimulate capillary formation, given that others have used higher concentrations in the same model [23]. It is important to note that majority of the infiltrated cells were not CD31-positive cells and likely to be inflammatory cells [24]. It is also important to note that the Matrigel plug model is in fact a better model for assessing cell invasion and invasive migration. This model is commonly used to investigate tumor angiogenesis, mimicking the cancer milieu [25,26]. Moreover, FGF-2 can promote cancer cell invasion [26] and metastasis [27,28]. Whether the cellular infiltration response in vivo induced by TRAIL or FGF-2 is an angiogenic (controlled cellular infiltration) or an invasive response (uncontrolled/aberrant cellular infiltration) is still unclear and requires more investigation.

Overall, in the present study, we find that TRAIL is more effective at promoting pro-angiogenic responses in vitro than VEGF-A at 10 ng/mL, but has a comparable response to FGF-2 at the same concentration. Combination treatment significantly enhanced the migration phase of angiogenesis in vitro. While a true angiogenic response with each treatment could not be determined in our model in vivo, our findings demonstrate increased potential for TRAIL-induced angiogenesis. Ultimately, further studies are warranted to determine whether TRAIL’s angiogenic response in vitro is replicated in vivo and whether TRAIL could be targeted to inhibit or activate angiogenesis.

## 4. Materials and Methods

### 4.1. Reagents

Matrigel (Matrigel^®^ matrix basement membrane growth factor reduced) was sourced from Corning (New York City, NY, USA). Human recombinant TRAIL was purchased from R&D Systems (Minneapolis, MN, USA). Human recombinant VEGF-A and FGF-2 were purchased from Sigma-Aldrich (St. Louis, MO, USA).

### 4.2. Tissue Culture

The human microvascular endothelial (HMEC) cell line has been previously described [4] and obtained from the Centers for Disease Control (Atlanta, GA, USA; MTA M1224I). Cells were cultured in MCDB 131 medium (Life Technologies, Carlsbad, CA, USA) supplemented with 10% foetal bovine serum, penicillin (5 U/mL) streptomycin (5 μg/mL), 200 mM l-glutamine, 100 μg/mL epidermal growth factor (EGF) and 200 μg/mL hydrocortisone. Cells were maintained at 37 °C in a humidified atmosphere of 5% CO_2_.

### 4.3. Total Cell Counts

HMEC-1 were seeded in a 96-well titre plate at a density of 6000 cells/well in quadruplicate in 200 μL 10% serum-containing MCDB 131 medium for 24 h. Cells were serum-arrested for 24 h and, unless indicated, were treated with TRAIL (10 ng/mL), FGF-2 (10 ng/mL), VEGF-A (10 ng/mL) or a combination of the three factors (each at 10 ng/mL). Total cell counts were assessed 24 and/or 48 h after treatment using a hemocytometer. Experiments were repeated at least three times.

### 4.4. In Vitro Migration Assay

HMEC-1 were grown in 6-well titre plates. Cells were serum-arrested at 80% confluency. After 24 h, a sterile P200 pipette tip was used to perform a scratch as previously described [29,30]. Briefly, cells were washed once with sterile 1× PBS followed by the addition of fresh serum-free media (2 mL/well), with or without TRAIL, FGF-2 and VEGF-A (10 ng/mL of growth factor individually or in combination). Images of the denuded area (4× magnification) were captured at room temperature using a DP71 Camera, DP Software and an Olympus IX71 microscope (Olympus Life Sciences, Shinjuku, Tokyo, Japan) 24 h after treatment. Four images/well were taken, and the percentage of cellular regrowth within the denuded zone was assessed by morphometric analysis using Adobe Photoshop Software. Experiments were repeated at least three times.

### 4.5. In Vitro Tubule Assay

Thirty thousand cells/well of serum-arrested HMEC-1 were seeded onto solidified Matrigel (100 μL) in a 96-well titre plate in 100 μL of serum-free medium [4]. At the time of seeding, cells were treated with TRAIL (10 ng/mL), VEGF-A (10 ng/mL), FGF-2 (10 ng/mL) or a combination of the three factors (each at 10 ng/mL). After 3 h, images of tubules (4 random fields of view at 10× magnification) were captured at room temperature using a DP71 Camera, DP Software and an Olympus IX71 microscope (Olympus Life Sciences, Shinjuku, Tokyo, Japan). The average length (μm) of tubules was quantified using NIH ImageJ software. For each image, 40 tubules were selected at random for analysis. Experiments were repeated three times.

### 4.6. Western Blotting

HMEC-1 were grown in 6-well titre plates. Cells were serum-arrested at ~80%–90% confluency for 24 h. Cells were then treated with TRAIL (10 ng/mL), VEGF-A (10 ng/mL) and FGF-2 (10 ng/mL) for 15 min, followed by total protein isolation using 1× SDS-sample buffer (62.5 mM Tris-HCl, pH 6.8, 10% glycerol, 2% SDS, 50 mM dithiothreitol and 0.1% bromophenol blue), as previously described [4]. Lysates were boiled for 5 min, followed by separation using 10-well Bolt™ 4%–12% Bis-Tris Plus Gels (Thermo Fisher Scientific, Waltham, MA, USA). The iBlot Gel Transfer System (Life Technologies, Carlsbad, CA, USA) was used to transfer proteins onto nitrocellulose membranes. Membranes were blocked in 5% bovine-serum albumin (in 1× PBS-Tween-20) for 1 h and then incubated overnight at 4 °C with rabbit polyclonal phospho-p42/44 MAPK(ERK1/2) antibody (1:5000; Cell Signaling Technology, Danvers, MA, USA) or 42/44 MAPK(ERK1/2) (1:3000; Cell Signaling Technology, Danvers, MA, USA). Proteins were detected with horseradish peroxidase-conjugated secondary anti-rabbit antibodies (1:3000; Dako, Glostrup, Denmark) and visualized by chemiluminescence (Amersham, GE Healthcare Life Sciences, Logan, UT, USA).

### 4.7. Matrigel Plug Assay

Eight- to twelve-week-old female C57Bl6 mice were handled according to the guidelines of the Sydney Local Health District Animal Welfare Committee Sydney, Australia (Protocol ID Number 2014-027C; approved March 2015). Mice were injected subcutaneously in their flank with 500 μL of Matrigel containing TRAIL, VEGF-A and/or FGF-2 at indicated concentrations. Plugs were resected 14 days later and fixed in 10% paraformaldehyde. Hematoxylin and eosin (H&E) stained images were captured at room temperature with an AxioCam HRc camera and Axio Imager Z2 Microscope (Carl Zeiss, Oberkochen, Germany). Images were acquired using Zen Pro software (Carl Zeiss, Oberkochen, Germany) and converted to Tiff files for further analysis. Total cell infiltration into the plug was measured using the ImageJ counting tool (1 image at 4× magnification/plug) and expressed as the number of cells per total area. The identity of blood vessels was confirmed with CD31 (1:50; Abcam, Cambridge, UK) staining in sister sections, quantified using Image-Pro^®^ Premier software (Media Cybernetics, Washington, MD, USA). Briefly, thresholds for positive staining were determined and threshold brown staining quantified (3 images at 10× magnification/plug). Data were expressed as % CD31 stained area. For the representative images shown in Figure 4 and Figure 5, changes to contrast/brightness were applied equally across the entire image.

### 4.8. Statistics

GraphPad Prism Version 6.0 (GraphPad Software Inc., San Diego, CA, USA) was used to analyse all data. Statistical comparisons were performed using unpaired Student’s *t*-test or one-way ANOVA with Tukey’s post-test adjustment. The results were considered statistically significant when *p* < 0.05.
